# Spacing, Feedback, and Testing Boost Vocabulary Learning in a Web Application

**DOI:** 10.3389/fpsyg.2021.757262

**Published:** 2021-11-15

**Authors:** Angelo Belardi, Salome Pedrett, Nicolas Rothen, Thomas P. Reber

**Affiliations:** ^1^Faculty of Psychology, UniDistance Suisse, Brig, Switzerland; ^2^Department of Epileptology, University of Bonn, Bonn, Germany

**Keywords:** distance education, distance learning, online learning, web application, memory, language learning, vocabulary learning, CALL (Computer Assisted Language Learning)

## Abstract

Information and communication technology (ICT) becomes more prevalent in education but its general efficacy and that of specific learning applications are not fully established yet. One way to further improve learning applications could be to use insights from fundamental memory research. We here assess whether four established learning principles (spacing, corrective feedback, testing, and multimodality) can be translated into an applied ICT context to facilitate vocabulary learning in a self-developed web application. Effects on the amount of newly learned vocabulary were assessed in a mixed factorial design (3×2×2×2) with the independent variables Spacing (between-subjects; one, two, or four sessions), Feedback (within-subjects; with or without), Testing (within-subjects, 70 or 30% retrieval trials), and Multimodality (within-subjects; unimodal or multimodal). Data from 79 participants revealed significant main effects for Spacing [*F*(2,76) = 8.51, *p* = 0.0005, $\eta_{p}^{2}=0.18$] and Feedback [*F*(1,76) = 21.38, *p* < 0.0001, $\eta_{p}^{2}=0.22$], and a significant interaction between Feedback and Testing [*F*(1,76) = 14.12, *p* = 0.0003, $\eta_{p}^{2}=0.16$]. Optimal Spacing and the presence of corrective Feedback in combination with Testing together boost learning by 29 percentage points as compared to non-optimal realizations (massed learning, testing with the lack of corrective feedback). Our findings indicate that established learning principles derived from basic memory research can successfully be implemented in web applications to optimize vocabulary learning.

## Introduction

Information and communication technology (ICT) changes how we access information and the way we learn. Smartphones, tablets, and desktop-computers become ubiquitous in living rooms and classrooms, transforming how learners of all ages perceive and interact with learning material. Identifying how ICT may improve learning is vital to ensure successful adaptation of educational practices for the digital age (Sung et al., 2016). In the current work, we investigate this general question by addressing the following specific research gaps: (1) Can some of the best researched learning principles originating from basic memory research be applied to optimize computer-assisted learning environments? (2) How do these learning principles interact? We do that in the setting of vocabulary learning because it is a central task in classes of foreign languages in schools. Vocabulary learning lends itself well to assess these questions because the transfer between basic memory research and its application seems rather close: vocabulary learning essentially entails long-term storage of memories for paired associates (i.e., a word and its associated translation in the foreign language), a well-researched phenomenon in basic memory research (Steinel et al., 2007).

A meta-meta-analysis in 2011 summarized 25 meta-analyses and found a small-to-moderate effect favoring the use of computer technology in the classroom to support teaching and learning, but also great variability among the results (Tamim et al., 2011). The efficacy of interactive learning applications to improve learning in classrooms is similarly promising and unclear (Sung et al., 2016). Some meta-analyses report beneficial effects initially that fade after 6–12 months (Cheung and Slavin, 2013; Sung et al., 2016). This fading of effects might represent an initial motivation boost when ICT is first introduced. As plans to invest more resources for ICT in classrooms emerge (Roediger and Pyc, 2012; Futuresource Consulting, 2016; European Commission, 2019), more research is needed that investigates not just whether but *how* ICT can be successfully applied in education. A comprehensive systematic review of meta-analyses compared more than 100 variables’ effects on achievement in higher education (Schneider and Preckel, 2017). ICT variables only had effects in the medium to lower ranges (Cohen’s *d* between 0.05 and 0.51), which might indicate that while ICT is applied in education, there is room for improvement about how this is done. The authors of that review also highlighted the shortage of controlled experiments on recent innovations in education. Such efforts could deepen our understanding of the variation of outcomes reported earlier and identify features of apps and learning situations contributing to more successful implementations of ICT in education.

One approach to scrutinize underlying mechanisms of ICT success is to take the perspective of a researcher interested in fundamental memory processes. While insights from memory research have often influenced the design of learning applications, especially in vocabulary learning, we think this perspective can further improve common educational practices in classrooms or learning applications if investigated systematically (Roediger and Pyc, 2012; Reber and Rothen, 2018). A commentary has been recently proposed to focus on four established learning principles known to facilitate learning in laboratory situations, which are also straightforward to implement in digital learning applications (Reber and Rothen, 2018).

Probably the most research-backed of the four principles is derived from the *spacing effect*. Spacing refers to splitting up the learning time into several short sessions and distributing them over time (Kornell et al., 2010; Carpenter et al., 2012). Learning is improved when we space out the learning time into separate distributed sessions, in contrast to cramming it into one session, also called massing (Cepeda et al., 2006; Benjamin and Tullis, 2010; Delaney et al., 2010).

A second principle concerns giving *corrective feedback* about mistakes in comparison with no feedback or simple right/wrong feedback (Metcalfe, 2017). From a cognitive perspective, corrective feedback leads to a “prediction-error” signal in the brain (Wilkinson et al., 2014), which catalyzes learning by switching brain regions relevant for long-term memory into a more receptive encoding rather than retrieval mode (Lisman and Grace, 2005; Greve et al., 2017).

The third principle builds on the *testing effect* (also test-enhanced learning or retrieval practice). When people have to reproduce or answer questions about the studied material, they remember more than when they study it repeatedly (Rowland, 2014). Better performance due to testing has been explained, on the one hand, by the *transfer-appropriate processing framework*, which posits that memory is better when learning and test situations are similar rather than different (Morris et al., 1977). That is, being able to recall information is more likely when recalling information was practiced in comparison with restudied. On the other hand, testing situations afford more effort, which may lead to deeper encoding of material according to the *desired difficulties framework* (Bjork, 1994; Bjork and Kroll, 2015).

Finally, presenting the learning material *multimodally*, i.e., to multiple senses simultaneously, benefits learning as well (Kast et al., 2007, 2011; Shams and Seitz, 2008). Multimodal presentation is inarguably closer to how we perceive the world and learn every day, without deliberate effort (incidental learning), than to present learning material for only one sensory channel. Furthermore, e.g., audio-visual presentations of learning materials recruit larger regions of the brain –namely, the ones processing auditory and visual information – as compared to unimodal presentations (auditory or visual stimuli alone). These “many routes” (Bjork, 1975) by which a stimulus is processed for encoding are thought to facilitate retrieval by making use of redundant information stored in distributed brain regions (Murray and Sperdin, 2010).

While extensive data on these four principles exist, few studies assessed how they interact (Weinstein et al., 2018). A notable exception looked at the interaction between spacing and testing: in a word pair learning task, testing improved learning success and this effect was even higher when the learning time was spaced beyond mere addition of the main effects (Cull, 2000).

This is also interesting considering that popular language learning and general learning tools available online already implement some learning principles we investigated: Duolingo,^1^ Rosetta Stone,^2^ Memrise,^3^ Anki,^4^ and Quizlet,^5^ for example, all implement testing, feedback, and multimodality in some way or another. The flashcard-style learning applications Anki and Quizlet both further implement spacing based on the so-called Leitner system, an algorithm to space and prioritize flash-cards (Godwin-Jones, 2010). Duolingo applies a self-developed procedure for spaced repetition using Half-Life Regression (Settles and Meeder, 2016). Please note that what we mean in the context of this manuscript by “spacing” is slightly different from “spacing” in the Leitner system. We refer to spacing of individual learning sessions, whereas the mentioned spacing algorithms refer to the scheduling of individual learning items within and across individual learning sessions.

The above learning principles were mostly researched using traditional learning methods (no use of ICT) in laboratory or classroom settings. The purpose of this study is to investigate whether these principles also improve learning efficiency with a web application in a home environment. A further aim is to explore the pairwise interactions between these principles. Our main research question (RQ1) was: “Can established learning principles be used to optimize learning of vocabulary with a web application?” Consequently, our hypotheses were: learning success, as measured in a cued recall test, is improved when: (a) the time spent learning is spaced vs. massed (H1), (b) corrective feedback is given vs. no feedback is given (H2), (c) more retrieval trials are presented for a specific word pair (H3), and (d) stimuli were presented multimodally vs. unimodally (H4). Our second research question (RQ2) was: “Are there any significant pairwise interactions between these principles?” To approach these questions, we implemented a web application that allows for independent variation of presence or absence and/or parametrization of all four learning principles. German-speaking participants used the app to learn Finnish language vocabulary, and we tested their recall 2 days after their last learning session.

## Materials and Methods

### Design

Our study was a 3 × 2 × 2 × 2 mixed factorial design with independent variables *Spacing* (between-subjects, one, two, and four learning sessions), *Feedback* (within-subjects, with and without corrective feedback), *Testing* (within-subjects, proportions of retrieval and learning trials were 70/30 or 30/70%), and *Multimodality* [within-subjects, unimodal (visual only) and multimodal (audio-visual)]. The dependent variable was the proportion of correct translations recalled in the testing session. Additionally, we varied the independent variable *learning direction* (within-subjects) in the learning phase and *testing direction* (within-subjects) in the test phase.

### Participants

Participants were recruited among friends and acquaintances of the students in a class on experimental research in the bachelor’s program in psychology, conducted at UniDistance Suisse in the autumn semester of 2018. Psychology students acted as experimenters (*N*_*e*_ = 22) and recruited *N*_*p*_ = 87 participants. Participants received no compensation, but a small thank-you gift was made by some of the experimenters. Participants gave written informed consent.

We analyzed data from 79 participants [43 female, 30 male, six undisclosed gender; age ranged between 16 and 77 years (*M* = 39.7, SD = 15.5)] after excluding 8 participants according to the following criteria: 3 had not completed the learning phase, three had not adhered to the scheduled gaps between sessions required for proper operationalization of the spacing factor, one had a long gap (over 20 min) during the testing session, and for one participant, age data were missing.

All participants were either native or advanced German speakers (73 natives, four near-native, one proficient, and one advanced). None of the participants reported any previous knowledge of the Finnish language or any closely related language, such as Hungarian or Estonian.

Fourteen participants held a bachelor’s degree, 21 a master’s degree, 25 had finished an apprenticeship, eight held a higher education entrance qualification, five had finished compulsory education, and six reported another form of education or were still in school.

### Materials

#### Stimuli

We used 48 Finnish-German word pairs as stimuli, gathered from various lists of frequently used words in Finnish and English, lyrics of Finnish pop songs, and words from a Finnish online dictionary. We filtered an initial list of 250 words and removed Finnish words that seemed too similar to a German word, ambiguous terms, compound words, interrogatives, personal pronouns, and terms that were subjectively too complicated or too simple. This resulted in 214 stimuli, out of which the final set of 48 word pairs was selected randomly. A list of all stimuli is available in the supporting materials online repository at https://osf.io/djxmr. Audio files for the stimuli were created with the text-to-speech software Balabolka (v. 2.14.0.676, Ilya Morozov).

#### Learning Application

For the learning phase, we developed a web application written in the R programming language (R Core Team, 2018), and we used the “shiny” (Chang et al., 2018) and “ShinyPsych” (Steiner et al., 2018) packages. We hosted the applications with the open source version of “Shiny Server” (v. 1.5.9.923, RStudio, Boston, MA, United States) on a virtual server running Linux Debian (v. 4.9.110). The participants could use the app by navigating to an URL linked to our server and start learning sessions using a personalized username and password. The app recorded all data on the webserver.

For the implementation of the between-subjects factor Spacing, we kept the overall learning time equal for all participants but split it into either (a) one learning session of 80 min, (b) two sessions of 40 min, or (c) four sessions of 20 min. The sessions were further split into 20-min learning blocks. Thus, every participant conducted four 20-min learning blocks altogether. The levels of the variable Spacing (one, two, or four sessions) were equally distributed among the experimenters, but the participant allocation to the levels was not done entirely at random: the rigid scheduling of several sessions would have made it impossible for some to participate. Thus, the participants’ preferences were considered in regard to having two, three, or five sessions with the experimenter (one, two, or four learning sessions plus one testing session). The participants did not know any specifics about the experiment or study procedure at the point on which the sessions were scheduled. The overall procedure was only explained to them after scheduling the sessions, during the first learning session.

The 48 word pairs were assigned to the 16 factor-combinations of the three main within factors (Feedback, Testing, and Multimodality) and learning direction. This assignment was randomized for each participant but was fixed during the whole learning phase of the participant.

**FIGURE 1:**
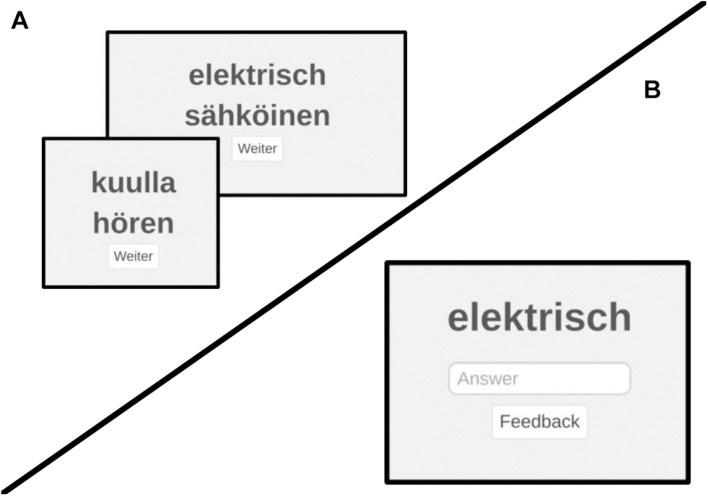
Screenshots of learning and retrieval trials in the web app. Screenshots showing examples of learning trials **(A)** and retrieval trials **(B)** as they were displayed to participants in the web applications. Translation for German terms: “Weiter” = “continue.”

Testing varied in the proportion of *learning* vs. *retrieval* trials. *Learning trials* entailed the presentation of a word pair in both languages and *retrieval trials* entailed presentation of one word as cue (German or Finnish) and an input field in which participants were prompted to input the translation of the word (see Figure 1). Each trial was either a learning or retrieval trial. Among all trials of one word pair, the proportion of retrieval trials and learning trials was set to either “70% retrieval trials and 30% learning trials” or “30% retrieval trials and 70% learning trials.”

**FIGURE 2:**
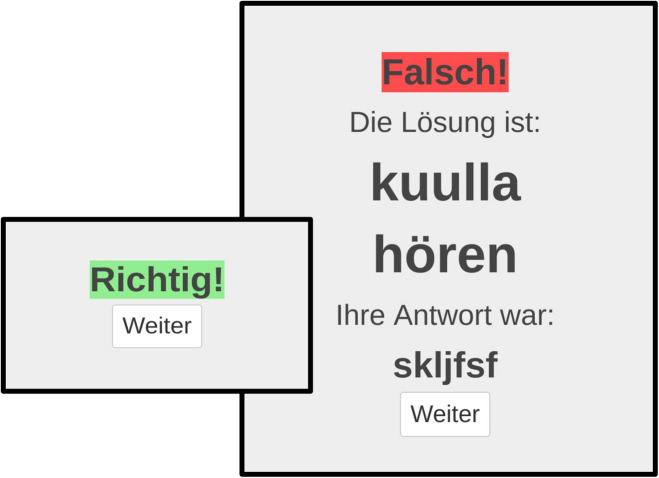
Screenshots showing feedback screens as they were displayed to participants in the learning application. On the left side is an example of the feedback for correct answers, and on the right side is an example of corrective feedback in case of a wrong response, which also shows the complete word pair and the wrong answer the participant gave. Translation for German terms: “Falsch” = “wrong,” “Richtig” = “correct,” “Die Lösung ist” = “the solution is,” “Ihre Antwort war” = “your answer was,” “Weiter” = “continue.”

Corrective feedback was provided for some translations, but not for others, and entailed showing the correct solution along with the cue word and the answer given by the participant, after an incorrect answer to a retrieval trial. If the answer was correct, feedback entailed the presentation of the word “correct” and the participant could proceed to the next trial (see Figure 2).

Multimodality entailed trials with multimodal vs. unimodal presentation. For multimodal (audio-visual) stimulus presentation, a word was displayed in either German or Finnish, while an audio file of the word spoken by a computer voice was played simultaneously in the same language. In learning trials, the audio recording was played only for the word displayed on top of the screen, not for the translation in the other language shown below. In unimodal trials, no audio recording was played.

We controlled for potential effects of learning direction. A word pair could either be learned in the direction from L1 (German) to L2 (Finnish) or the other way around (*L2-to-L1*). In learning trials, the first word was at the top of the screen, and its translation was below. In retrieval trials, the first word was at the top, and the input field into which the participants could enter the translation was below.

#### Test Application

The test application was used during the final testing session and displayed only retrieval trials. We varied the independent variable *testing direction*: each of the 48 word pairs was tested once in either direction (L1-to-L2 and L2-to-L1), resulting in 96 trials. Participants received no feedback on individual trials.

#### Questionnaires

We assessed sociodemographic information and motivation with a questionnaire created in LimeSurvey (v.3.14.3+180809, LimeSurvey GmbH, Hamburg, Germany). Motivation was measured with the Questionnaire on Current Motivation (QCM) in its German version “Fragebogen zur Erfassung aktueller Motivation in Lern- und Leistungssituationen” (Rheinberg et al., 2001). For the German QCM, an internal consistency between Cronbach’s alpha 0.66 and 0.90 was found in different samples, and convergent validity was assessed by correlating QCM scales with subscales from another instrument to assess motivational factors (the Multi-Motive-Grid; Schmalt et al., 2000; correlations of *r* = 0.29, *p* < 0.05 and *r* = −0.30, *p* < 0.05) (Rheinberg et al., 2001).

### Procedure

For each learning and testing session, one experimenter met individually with one participant at a time. Experimenters followed a written guideline (available in the supporting materials online repository at https://osf.io/djxmr). The experiment was either conducted at the experimenter’s or the participant’s home, and participants could use their own computer or one provided by the experimenter. There was an exception for four experimenters (and thus 16 participants), who tested their participants without being physically present. Instead, they kept contact with the participants *via* video call on an additional device during the experiment. Whether participants were tested remotely or not had no effect on the conclusions of the experiment, as analyses excluding these participants were virtually identical to the main analyses presented in the Results section (Supplementary Table 1).

The experiment started with the online questionnaire. Next, the participants began learning with the web application in their first study session. The first screen contained information on how to interact with the application and a query to check the audio settings.

Depending on the level of Spacing, the participants performed multiple 20-min blocks in the same learning session and could take short breaks in-between (about 5–10 min). Within each 20-min block, there were three phases of equal duration during which a set of 16 word pairs was learned, one word for each of the 8 within-subject factor-combinations, in both learning directions. The three sets were presented in the same order in all 20-min blocks.

For each trial, a word pair was randomly chosen from the active set of 16 word pairs. If the word pair was chosen for the first time, it was presented as learning trial; otherwise, it was presented as learning or retrieval trial with a probability according to the Testing condition. Learning trials proceeded by button press or mouse click; retrieval trials by submitting a response *via* keyboard, followed by feedback depending on the condition.

Overall, all participants learned the same 48 word pairs and had a total learning time of 80 min. Due to randomized presentation of word pairs, the number of trials per word pair and participant varied (*M* = 22.3, SD = 0.687), but ANOVA showed that while there were slight differences in the number of trials between the factor conditions, those are unlikely to account for our results (see Supplementary Tables 3, 4).

Between learning sessions and between the last learning session and the testing session, a gap of 2 days was planned and the actual mean gap time ranged from 42.8 to 77.2 h between subjects (Med = 69.7, IQR = 22.8). We will refer to the gap between individual learning sessions as *inter-study interval* (ISI) and to that between the last learning session and the testing session as *retention interval* (Cepeda et al., 2006). In the testing session, participants conducted a cued recall test of all learned translations using the testing application. The 96 trials were presented in a randomized order.

## Results

### Learning Principles

**FIGURE 3:**
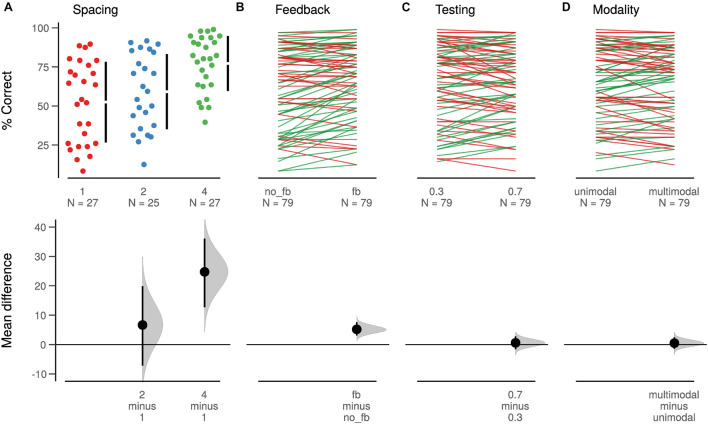
Estimation plots for the learning principles *via* Cumming plots. Upper row shows individual participant data in a swarmplot for unpaired data (**A**, Spacing) and a slopegraph for paired data (**B**, Feedback, **C**, Testing, and **D** Modality). For unpaired data, the mean ± SD are shown as gapped lines. Lower row shows unpaired or paired mean differences as a bootstrap sampling distribution, with the dot indicating the mean difference and the ends of the error bars the 95% confidence interval.

**FIGURE 4:**
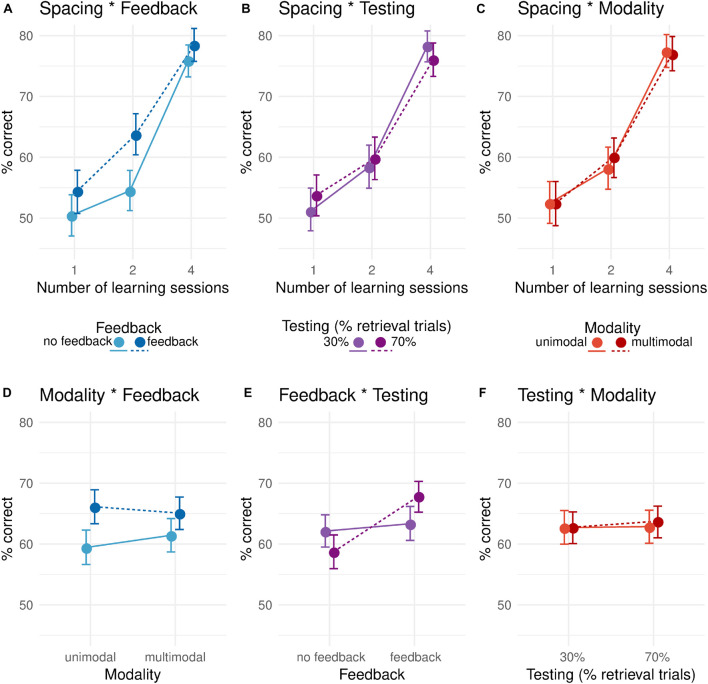
Pairwise interaction plots between learning principles. **(A–C)** Interactions with between-subjects factor Spacing. **(D–F)** Interactions between the three within-subjects factors Feedback, Testing, and Modality. Values are offset horizontally to avoid over-plotting of error bars. Error bars indicate non-parametrically bootstrapped 95% confidence intervals.

To assess the effects of the four learning principles on recall, we conducted a four-way 3×2×2×2 mixed-design ANOVA with factors Spacing (one, two, or three learning sessions), Feedback (with or without feedback), Testing (70% retrieval trials or 30%), and Multimodality (unimodal or multimodal; Figures 3, 4). Dependent variable was the proportion of correctly recalled words during the testing session.

We found a main effect for the factor Spacing [*F*(2,76) = 8.51, *p* = 0.0005, $\eta_{p}^{2}=0.18$]. In support of H1, participants in which learning was distributed the most (four sessions) had the highest recall performance (*M* = 77.2, SD = 29). Performance was intermediate in participants who learned during two sessions (*M* = 59.1, SD = 35.3) and lowest in the massed learning condition (1 session; *M* = 52.5, SD = 37; see Figure 3A). The ANOVA also revealed a main effect of corrective Feedback [*F*(1,76) = 21.38, *p* < 0.0001, $\eta_{p}^{2}=0.22$; see Figure 3B]. As hypothesized (H2), the recall performance was higher (*M* = 65.6, SD = 35) on translations to which corrective feedback was provided in the learning phases than on translations without feedback during learning (*M* = 60.4, SD = 35.9).

The main effects for the factors Testing (H3) and Multimodality (H4) were both insignificant [*F*_Testing_(1,76) = 0.31, *p*_Testing_ = 0.58, $\eta_{p\mathrm{Testing}}^{2}=0.004$; *F*_Multimodality_(1,76) = 0.26, *p*_Multimodality_ = 0.61, $\eta_{p\mathrm{Multimodality}}^{2}=0.003$; Figures 3C,D]. One potential explanation for the absence of an effect of testing may arise from considering the two-way interactions of the ANOVA (RQ2). Here, a significant interaction between the factors Testing and Feedback was found [*F*(1,76) = 14.12, *p* = 0.0003, $\eta_{p}^{2}=0.16$; Figure 4E]. Recall performance was higher in the feedback vs. no-feedback condition only when combined with a high rate of retrieval trials (0.7) administered during learning. No such difference was found for a low rate of retrieval trials. Thus, as there is no main effect of Testing, it appears that Testing, nevertheless, improved learning performance, but only in situations when Testing was combined with corrective Feedback. No other two-way interaction reached significance. For an overview of all effects in the ANOVA, see Table 1.

**TABLE 1:** ANOVA learning principles.

Effect	*df*	*MSE*	*F*	$\eta_{p}^{2}$	*p*-Value
Spacing	2, 76	4143.08	8.51	0.18	0.0005
Modality	1, 76	156.01	0.26	0.003	0.61
Spacing:Modality	2, 76	156.01	0.5	0.01	0.61
Testing	1, 76	185.77	0.31	0.004	0.58
Spacing:Testing	2, 76	185.77	1.85	0.05	0.16
Feedback	1, 76	204.98	21.38	0.22	<0.0001
Spacing:Feedback	2, 76	204.98	3.11	0.08	0.05
Modality:Testing	1, 76	87.46	0.37	0.005	0.54
Modality:Feedback	1, 76	125.67	2.83	0.04	0.1
Testing:Feedback	1, 76	178.79	14.12	0.16	0.0003

*We only report main effects and two-way interactions, because our research question RQ2 focused on pairwise interactions. df, degrees of freedom; MSE, mean-squared error; $\eta_{p}^{2}$, partial eta squared.*

Rather than merely looking at the statistical significance, we think that specifically in an applied context, it is crucial to consider effect sizes. Spacing led to 24.7 percentage points higher recall when participants learned in four spaced sessions instead of in one massed session. Corrective Feedback increased recall by 5.2 percentage points. Due to the combination of feedback and testing, recall gained another 5.8 percentage points. The optimal combination of factors levels was four learning sessions, feedback, and 70% retrieval trials. The observed means of our sample show that this combination and the one with 30% retrieval trials were at the top, with almost identical values of 78.2 and 78.7% correctly recalled words. The least efficient combination for learning consisted of one learning session, no feedback, and 70% retrieval trials and led to 49.5% recall. The difference between the observed best and worst combination was thus a boost of 29 percentage points.

One issue we had was that subjects were not entirely random assigned to the factor levels of Spacing. To assess a potential confound of Spacing with other subject-specific variables (e.g., motivation and interest), we conducted a one-way ANOVA of the factor Spacing on learning performance during the first block of the experiment on the retrieval trials only. This block was comparable with respect to Spacing for all participants. Here, a significant effect of Spacing was found [*F*(2,75) = 3.18, *p* = 0.047]. We then conducted the full factorial ANOVA (3 × 2 × 2 × 2 for the factors Spacing, Feedback, Modality, and Testing) also on performance in retrieval trials of the first block of learning only. Here, the factor Spacing, again, was significant [*F*(2,66) = 5.32, *p* = 0.007]. The factor Feedback [*F*(1,66) = 60.45, *p* < 0.001] as well as the interaction of Testing and Feedback [*F*(1,66) = 8.26, *p* = 0.005] were significant. These results are similar to the main analysis above with performance of the final testing session as dependent variable. Together, these results are consistent with the notion of a confound of participant factors with the experimental factor of Spacing. The effects of Feedback and the interaction of Testing and Feedback, in contrast, are unaffected by this potential confound.

### Exploratory Analyses: Learning and Testing Direction

Each word pair was learned in one direction, either L1-to-L2 or L2-to-L1. Furthermore, since participants performed the recall task in both directions, we could also assess effects of testing direction and the interaction between learning direction and testing direction. This resulted in an additional ANOVA with six factors, adding learning direction and testing directions to the model.

**FIGURE 5:**
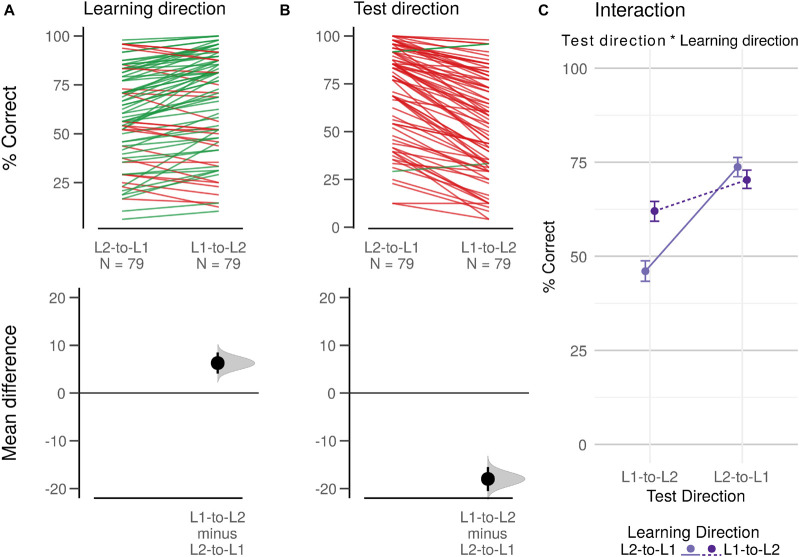
Estimation plots and interaction plot for learning and testing direction. Estimation plots for learning direction **(A)** and testing direction **(B)**. Upper row shows paired individual participant data in slopegraphs. Lower row shows paired mean differences as a bootstrap sampling distribution, with the dot indicating the mean difference and the ends of the error bars indicating the 95% confidence interval. **(C)** Interaction plot. Values are offset horizontally to avoid over-plotting of error bars. Error bars indicate bootstrapped 95% confidence intervals.

Learning direction had a substantial effect on recall performance [*F*(1,76) = 28.61, *p* < 0.0001, $\eta_{p}^{2}=0.27$, see Figure 5A], where the words which participants learned in the direction L1-to-L2 (*M* = 66.2, SD = 34.4) were recalled better than those in the direction L2-to-L1 (*M* = 59.9, SD = 36.3). Adding learning direction to the design features described above we observe a difference of 38 percentage points between best and worst combinations of features of the learning app (see Table 2). Regarding testing direction, recall performance was generally higher for the direction L2-to-L1 (*M* = 72.0, SD = 31.9) as compared to L2-to-L1 (*M* = 54.0, SD = 36.7, *F*(1,76) = 233.38, *p* < 0.0001, $\eta_{p}^{2}=0.75$; see Figure 5B).

**TABLE 2:** Proportions of correctly recalled word pairs in combinations of Spacing, Feedback, Testing, and Learning direction.

Feedback	Testing (%)	Learning direction	Spacing		
			1	2	4
No feedback	30	*L2-to-L1*	50.31	54.67	75.62
No feedback	70	*L2-to-L1*	**44.14**	48.67	71.91
No feedback	30	*L1-to-L2*	52.47	58.67	80.25
No feedback	70	*L1-to-L2*	54.94	56.00	75.93
Feedback	30	*L2-to-L1*	46.60	57.67	75.62
Feedback	70	*L2-to-L1*	54.94	63.00	74.38
Feedback	30	*L1-to-L2*	55.25	62.67	81.79
Feedback	70	*L1-to-L2*	61.11	71.67	**82.10**

*Proportions of correctly recalled word pairs in specific factor combinations over all participants. Each participant learned six of the 48 word pairs in each of the eight shown within-subjects factor combinations (Feedback × Testing × Learning direction). Minimal and maximal values are set in bold font.*

We further found an interaction between Learning direction and Testing direction [*F*(1,76) = 105.74, *p* < 0.0001, $\eta_{p}^{2}=0.58$; see Figure 5C]; words learned in the direction L2-to-L1 were recalled much better when the testing direction matched. For words learned in the direction L1-to-L2, the recall difference was much smaller and recall was actually higher when the testing direction did not match. The complete results table for this exploratory analysis is available in Supplementary Table 2.

### Covariates Age, Number of Trials, and Motivation

We checked the influence of the potential covariates age, number of trials, and motivational factors. Performance in cued recall tasks usually dwindles with higher age (Park et al., 1996). To control for potential age effects, we ran an ANCOVA adding age as a covariate to our main model of learning principles. There was a significant age effect [*F*(1,76) = 21.68, *p* < 0.0001, $\eta_{p}^{2}=0.23$], but the main results of the learning principles remained virtually identical when controlling for age.

The number of trials the participants saw during their learning sessions depended on how quickly they pressed the button to continue to the next trial (in learning trials) or entered their answers (in retrieval trials). Therefore, the number of trials varied substantially between subjects (*M* = 1071, SD = 386, range: 338–2213). To check whether this had an effect on recall itself and whether it influenced the findings of our main model, we ran another ANCOVA, adding number of trials as a covariate. While we found a significant effect of number of trials [*F*(1,73) = 7.56, *p* = 0.008, $\eta_{p}^{2}=0.09$], the other results remained similar to the main model.

Before the first learning session, we assessed motivation related to the learning task. Two of the motivational factors in the questionnaire we used, namely, fear of failure and success seeking, are related to tasks described as question-led fact learning, a definition into which our vocabulary learning task seems to fit (Rheinberg et al., 2001). We consequently ran two additional models, including each of these factors in turn as a covariate, but there were no significant effects of fear of failure [*F*(1,69) = 0, *p* = 0.95, $\eta_{p}^{2}<0.0001$] or success seeking [*F*(1,62) = 0.13, *p* = 0.72, $\eta_{p}^{2}=0.002$] and the general results were similar to those of the main model.

## Discussion

We investigated whether four learning principles (Spacing, Feedback, Testing, and Multimodality) derived from fundamental memory research can be used to optimize a web-application in a real-world digital context for vocabulary learning. Varying the presence/absence or parameters of each of these principles independently, we find that Spacing and the presence of corrective Feedback and Testing together significantly boost learning by 29 percentage points. Our results hence demonstrate that informing the development of ICT applications with knowledge from basic memory research can significantly ameliorate their efficiency.

We found an increased recall of approximately 25 percentage points due to *Spacing*, which is in the medium range of what previous studies with vocabulary learning paradigms report (Bloom and Shuell, 1981; Cepeda et al., 2009; Nakata, 2015; Lotfolahi and Salehi, 2017). The range of reported spacing effects in studies with L2 vocabulary is rather large as effects between 13 and 35 percentage points have been reported. Of course, different study designs and learning intensity are likely origins of this variation. One study that used similar conditions to those in ours (3-day retention interval; fixed ISI of 2 days; four learning sessions; first learning session lasted about 30 min; 40 word pairs; computer-based flash-card app) found a difference of almost 50 percentage points in recall between the uniformly distributed and massed learning conditions (Cull, 2000). A likely reason for a higher effect of spacing than in our study is that they used uncommon-common L1 word pairs instead of L2 vocabulary and the learning time was not fixed. Our significant effect of Spacing, however, has to be taken with a grain of salt since participants were sometimes non-randomly assigned to the levels of Spacing to due to scheduling constraints. A potential confound of Spacing with unknown participant factors cannot be ruled out.

In comparison with previous studies, we found a rather small benefit of giving corrective *Feedback* to improve vocabulary learning (5.2 percentage points higher recall for feedback vs. no feedback). It seems noteworthy that only few comparable studies exist that report the difference between corrective feedback and no feedback conditions in vocabulary learning experiments. One such study assessing five different feedback conditions did not report a significant effect (Pashler et al., 2005), while another reported increases in recall performance by immediate feedback as 11 and 18 percentage points (Metcalfe et al., 2009).

In our results, *Testing* influenced performance in an interaction together with *Feedback*. Participants could only profit from retrieval trials when they received feedback. This interaction was discussed already by Roediger and Butler (2011), though they reported that testing was often effective even when no feedback is given. To explore this further, one could also incorporate more levels for each factor, for example an option with simple right/wrong feedback (non-corrective) or with a rewrite variant, where subjects have to write out the correct answer directly after they got the corrective feedback. This might lead to deeper processing of the feedback.

To our surprise, *Multimodality* did not improve recall in our experiment. To put this finding into perspective, we can look at comparable computer-assisted language learning studies. These studies often investigated glosses and annotations in regard to multimodal presentation. One exemplary study found that combined text and image annotations outperformed those with text only but adding videos did not and emphasized the need to isolate the types of annotations in further studies and suggested the audio modality for further investigation (Chun and Plass, 1996). Further support for the use of images together with written definitions in vocabulary learning was found by another research group but their participants performed poorer when spoken text was added to written text instructions (Kim and Gilman, 2008). These researchers theorized that the problem might be that their participants were used to learning new vocabulary without knowing the pronunciation as is often the case for Korean native-speaking participants who learn English. Thus, the additional spoken word sounds might have distracted rather than helped. This might not be transferable to native German speakers in Switzerland who are used to focusing on the pronunciation of new vocabulary in language classes. A similar study reported no difference between text-only, image-only and combined text and image glosses (Yanguas, 2009). Overall, these mixed findings regarding multimodality are consistent with our results indicating no significant difference in the multimodality condition. The discussion of optimal learning environments by Moreno and Mayer (2007) might give us further clues about why we did not find an effect in our multimodal condition. They cautioned against delivering both verbal and non-verbal stimuli through the same modality (e.g., written word and images), since this could overload the learners’ cognitive capacity (Low and Sweller, 2014; Mayer and Pilegard, 2014).

Our findings add additional evidence for the advantage of the L1-to-L2 learning direction in a delayed recall test. In 2002, one study found that participants who had learned in the direction L1-to-L2 recalled less in an immediate test, but then performed marginally better one week later in comparison with those in the L2-to-L1 condition (Schneider et al., 2002). These results are in line with conclusions of another study that found the L1-to-L2 direction to be overall preferable when one learns for both, comprehension and production of the new vocabulary (Griffin and Harley, 1996). In line with our results, one study found an interaction of learning and testing direction: the apparently more difficult learning direction (L1-to-L2) helped in later recall only if the testing direction matched (Steinel et al., 2007). Together, these results may suggest that when a word pair is studied the easier way (L2-to-L1), participants have a hard time recalling and producing the word correctly in the difficult direction (L1-to-L2).

Based on a power analysis done in MorePower (Campbell and Thompson, 2012), our sample size was sufficient to find effects at or above a $\eta_{p}^{2}$ of 0.118 (Cohen’s *f* of 0.366) for the main effect and two-way interactions involving the three-level between-subjects factor and at or above a $\eta_{p}^{2}$ of 0.097 (Cohen’s *f* of 0.328) for main effects and two-way interactions involving only the two-level within-subject factors with a power of 0.8 at an alpha level of 0.05. Our study was thus adequately powered to detect medium to large effects for the main effects and two-way interactions. The reported significant findings in our main ANOVA (Table 1) were all well above the found thresholds, at $\eta_{p}^{2}$ of 0.16, 0.18, and 0.22. Further power analysis results are presented in Supplementary Table 5.

What are these findings telling us about how to develop learning apps? For any real-world application, the optimal implementation or combination of these learning principles might vary. In our experiment, three out of four learning principles improved later retrieval and one (Testing) did so only in combination with another (Feedback). Utilizing these principles in learning apps at all is a first step, which has been done by the developers of popular language learning apps. We can additionally gain more insights into how such learning principles interact with each other to make even better use of them, especially in an individually applied setting. Our approach to experimentally manipulate the presence and absence of learning principles furthermore allowed us to quantify the gains in memory due to individual learning principles and their interactions in an app.

## Conclusion

Three established learning principles, Spacing, corrective Feedback, and Testing in combination with corrective Feedback, improved vocabulary learning performance in the context of a web application with which German speakers learned Finnish. Recall improved by 29 percentage points when participants could use the learning principles. These findings support our notion that knowledge from fundamental memory research can inform the development of learning applications to improve them.

## Data Availability Statement

The datasets presented in this study can be found in online repositories. The names of the repository/repositories and accession number(s) can be found below: https://osf.io/djxmr/.

## Ethics Statement

The studies involving human participants were reviewed and approved by Ethical Review Committee of the Faculty of Psychology at UniDistance Suisse. The patients/participants provided their written informed consent to participate in this study.

## Author Contributions

AB: conceptualization, data curation, formal analysis, investigation, methodology, project administration, software, validation, visualization, writing – original draft, and writing – review and editing. SP: conceptualization, data curation, formal analysis, investigation, methodology, project administration, software, validation, and writing – review and editing. NR: conceptualization, resources, project administration, supervision, and writing – review and editing. TR: conceptualization, resources, formal analysis, investigation, methodology, project administration, software, validation, supervision, writing – original draft, and writing – review and editing. All authors contributed to the article and approved the submitted version.

## Conflict of Interest

The authors declare that the research was conducted in the absence of any commercial or financial relationships that could be construed as a potential conflict of interest.

## Publisher’s Note

All claims expressed in this article are solely those of the authors and do not necessarily represent those of their affiliated organizations, or those of the publisher, the editors and the reviewers. Any product that may be evaluated in this article, or claim that may be made by its manufacturer, is not guaranteed or endorsed by the publisher.

## Footnotes

1
www.duolingo.com

2
www.rosettastone.com

3
www.memrise.com

4
apps.ankiweb.net

5
www.quizlet.com
