# Correction: Spacing, feedback, and testing boost vocabulary learning in a web application

**DOI:** 10.3389/fpsyg.2025.1629682

**Published:** 2025-07-10

**Authors:** Angelo Belardi, Salome Pedrett, Nicolas Rothen, Thomas P. Reber

**Affiliations:** ^1^Faculty of Psychology, UniDistance Suisse, Brig, Switzerland; ^2^Department of Epileptology, University of Bonn, Bonn, Germany

**Keywords:** distance education, distance learning, online learning, web application, memory, language learning, vocabulary learning, CALL (Computer Assisted Language Learning)

In the published article, there were errors in various sections due to ambiguous phrasing regarding the reporting of changes in recall performance. Specifically, incorrect phrases regarding percent changes instead of absolute percentage points, which may be interpreted as relative percentage changes rather than the intended absolute percentage point differences (e.g., “improved by 29%” instead of “improved by 29 percentage points”).

A correction has been made to the **Abstract**. This sentence previously stated:

“Optimal Spacing and the presence of corrective Feedback in combination with Testing together boost learning by 29% as compared to non-optimal realizations (massed learning, testing with the lack of corrective feedback).”

The correct sentence appears below:

“Optimal Spacing and the presence of corrective Feedback in combination with Testing together boost learning by 29 percentage points as compared to non-optimal realizations (massed learning, testing with the lack of corrective feedback).”

A correction has been made to **Results**, *Learning Principles*, paragraph four. This sentence previously stated:

“Spacing led to 24.7% higher recall when participants learned in four spaced sessions instead of in one massed session. Corrective Feedback increased recall by 5.2%. Due to the combination of feedback and testing, recall gained another 5.8%.”

The correct sentence appears below:

“Spacing led to 24.7 percentage points higher recall when participants learned in four spaced sessions instead of in one massed session. Corrective Feedback increased recall by 5.2 percentage points. Due to the combination of feedback and testing, recall gained another 5.8 percentage points.”

A correction has been made to **Results**, *Learning Principles*, paragraph four. This sentence previously stated:

“The difference between the observed best and worst combination was thus a boost of 29%.”

The correct sentence appears below:

“The difference between the observed best and worst combination was thus a boost of 29 percentage points.”

A correction has been made to **Results**, *Exploratory Analyses: Learning and Testing Direction*, paragraph two. This sentence previously stated:

“Adding learning direction to the design features described above we observe a difference of 38% between best and worst combinations of features of the learning app (see Table 2).”

The correct sentence appears below:

“Adding learning direction to the design features described above we observe a difference of 38 percentage points between best and worst combinations of features of the learning app (see Table 2).”

A correction has been made to **Discussion**, paragraph one. This sentence previously stated:

“Varying the presence/absence or parameters of each of these principles independently, we find that Spacing and the presence of corrective Feedback and Testing together significantly boost learning by 29%.”

The correct sentence appears below:

“Varying the presence/absence or parameters of each of these principles independently, we find that Spacing and the presence of corrective Feedback and Testing together significantly boost learning by 29 percentage points.”

A correction has been made to **Discussion**, paragraph two. This sentence previously stated:

“We found an increased recall of approximately 25% due to Spacing, which is in the medium range of what previous studies with vocabulary learning paradigms report (Bloom and Shuell, 1981; Cepeda et al., 2009; Nakata, 2015; Lotfolahi and Salehi, 2017). The range of reported spacing effects in studies with L2 vocabulary is rather large as effects between 13 and 35% have been reported.”

The correct sentence appears below:

“We found an increased recall of approximately 25 percentage points due to Spacing, which is in the medium range of what previous studies with vocabulary learning paradigms report (Bloom and Shuell, 1981; Cepeda et al., 2009; Nakata, 2015; Lotfolahi and Salehi, 2017). The range of reported spacing effects in studies with L2 vocabulary is rather large as effects between 13 and 35 percentage points have been reported.”

A correction has been made to **Discussion**, paragraph two. This sentence previously stated:

“One study that used similar conditions to those in ours (3-day retention interval; fixed ISI of 2 days; four learning sessions; first learning session lasted about 30 min; 40 word pairs; computer-based flash-card app) found a difference of almost 50% in recall between the uniformly distributed and massed learning conditions (Cull, 2000).”

The correct sentence appears below:

“One study that used similar conditions to those in ours (3-day retention interval; fixed ISI of 2 days; four learning sessions; first learning session lasted about 30 min; 40 word pairs; computer-based flash-card app) found a difference of almost 50 percentage points in recall between the uniformly distributed and massed learning conditions (Cull, 2000).”

A correction has been made to **Discussion**, paragraph three. This sentence previously stated:

“In comparison with previous studies, we found a rather small benefit of giving corrective Feedback to improve vocabulary learning (5.2% higher recall for feedback vs. no feedback).”

The correct sentence appears below:

“In comparison with previous studies, we found a rather small benefit of giving corrective Feedback to improve vocabulary learning (5.2 percentage points higher recall for feedback vs. no feedback).”

A correction has been made to **Discussion**, paragraph three. This sentence previously stated:

“One such study assessing five different feedback conditions did not report a significant effect (Pashler et al., 2005), while another reported increases in recall performance by immediate feedback as 11 and 18% (Metcalfe et al., 2009).”

The correct sentence appears below:

“One such study assessing five different feedback conditions did not report a significant effect (Pashler et al., 2005), while another reported increases in recall performance by immediate feedback as 11 and 18 percentage points (Metcalfe et al., 2009).”

A correction has been made to **Conclusion**. This sentence previously stated:

“Recall improved by 29% when participants could use the learning principles.”

The correct sentence appears below:

“Recall improved by 29 percentage points when participants could use the learning principles.”

The original article has been updated.

