# Supplementary material for: Spacing, Feedback, and Testing Boost Vocabulary Learning in a Web Application
Source: Front Psychol. 2021 Nov 15;12:757262. doi: 10.3389/fpsyg.2021.757262 (PMC8638698; doi:10.3389/fpsyg.2021.757262)
Supplement: Supplementary file 1 [file Presentation_1.pdf]

UNIVERSITÄRE FERNSTUDIEN SCHWEIZ

Anleitung für Versuchsleiter

Dies ist eine Anleitung zur Durchführung des Experiments für das Projekt «Kognitive Lernprinzipien» im Modul 8: Methoden III: Experimentelle Übungen.

Terminfindung

Für das Experiment werden je nach Zuordnung der UV1 zwei bis fünf Sitzungen mit den Vpn vereinbart. Diese finden jeweils mit einem zeitlichen Abstand von zwei Tagen statt. Die Uhrzeit muss nicht identisch sein. Zuerst gibt es eine, zwei, oder vier Lernphasen, bei denen jeweils die ***Lernapp*** gestartet wird. Bei der letzten Sitzung, der Testphase, wird die ***Testapp*** gestartet.

Je nach UV1 sieht die Testung insgesamt wiefolgt aus:

Mit einer Lernsitzung:
=> 1., 2., 3. und 4. Lernblock, je 20 Minuten
=> zwei Tage Pause
=> Testblock

Mit zwei Lernsitzungen:
=> 1. und 2. Lernblock, je 20 Minuten
=> zwei Tage Pause
=> 3. und 4. Lernblock, je 20 Minuten
=> zwei Tage Pause
=> Testblock

Mit vier Lernsitzungen:
=> 1. Lernblock, 20 Minuten
=> zwei Tage Pause
=> 2. Lernblock, 20 Minuten
=> zwei Tage Pause
=> 3. Lernblock, 20 Minuten
=> zwei Tage Pause
=> 4. Lernblock, 20 Minuten
=> zwei Tage Pause
=> Testblock

Materialien

Zum Durchführen des Experiments werden folgende Dokumente und Materialien benötigt. Bitte bereiten Sie diese alle vor der ersten Sitzung vor. Überprüfen Sie auch möglichst jeweils vor den Sitzungen die Webapp, die sie benötigen werden. Also ob Sie die Webapplikationen grundsätzlich aufrufen können.

- **Anleitung für Versuchsleiter** (dieses Dokument)
- **Tabelle «Experimentdaten und Protokoll»** (individuelles Dokument für jede Versuchsperson. Jede testleitende Person erhält hierzu individuelle PDF-Dokumente. Enthält Versuchspersonennummer und Passwort für die Webapps und Platz für Notizen während der Testungen. Wichtig: Aussergewöhnliches festhalten, damit Abweichungen vom Experimentverlauf später nachvollzogen werden können. Z.B. Unterbrechungen, Probleme mit den Apps).
- **Computer/Notebook** mit Internetzugang und funktionierenden Kopfhörern oder Lautsprechern
- **Startseite** mit den Links zur ***Lernapp*** und ***Testapp*** und zum Prä-Fragebogen: http://neurocog.fernuni.ch/lernprinzipien
- Kopie der **Einverständniserklärung** (für jede Vpn zweimal ausdrucken) https://moodle.fernuni.ch/mod/resource/view.php?id=108448

Ablauf

1 Erste Lernphase / erste Sitzung

Dauer: 20 Minuten Einführung, dann 1, 2 oder 4 Lernblöcke à 20 Minuten (je nach UV1)

1.1 Erklärung Experiment und Einverständniserklärung

Sie beginnen die erste Sitzung mit einer kurzen Erklärung an die Versuchsperson:
Es geht beim Experiment darum, mit einer Webapplikation Vokabeln einer Fremdsprache (Finnisch) zu lernen. Dabei gibt es Lerndurchgänge, bei denen jeweils einfach ein Wort auf Deutsch und Finnisch angezeigt wird, und Abrufdurchgänge, bei denen das Wort nur auf Deutsch oder nur auf Finnisch angezeigt wird und die Versuchsperson die Übersetzung selbst eintragen muss.
Weiter kommt man immer mit der [ENTER] Taste oder mit einem Klick auf den Button unter der Abfrage bzw. unter den angezeigten Wörtern. Man kann auch weiter klicken, ohne eine Antwort einzugeben. Ein Lernblock dauert 20 Minuten. Wenn ein Block beendet ist, erscheint eine Seite mit weiteren Hinweisen in der Webapp.

Nach der Instruktion geben Sie der Versuchsperson die Einverständniserklärung, damit sie diese durchlesen kann. Beantworten Sie allfällige Fragen der Versuchsperson. Wenn die Versuchsperson die Erklärung komplett gelesen und verstanden hat und ihr Einverständnis zur Teilnehme am Experiment geben möchte, muss sie die Einverständniserklärung ausfüllen und unterschreiben. Geben Sie der Versuchsperson zusätzlich eine Kopie der Einverständniserklärung ab, die diese bei sich behalten kann. Bitte bewahren Sie die ausgefüllten Einverständniserklärungen auf und geben Sie diese am Schluss der Untersuchung den Betreuern Ihrer Gruppe ab.

1.2 Fragebogen Demografie

Nun rufen Sie über die Startseite (http://neurocog.fernuni.ch/lernprinzipien) den Prä-Fragebogen auf. Geben Sie Vpn-Nr und Passwort ein und lassen Sie die Vpn den Fragebogen vollständig ausfüllen.

1.3 Lernblöcke

Kopieren Sie das Passwort für die App, sodass Sie es schnell zur Hand haben. Dann gehen Sie via Startseite (http://neurocog.fernuni.ch/lernprinzipien) zur ***Lernapp***, wo Sie zunächst die Vpn-Nr und das Passwort der Vpn eingeben. Im nächsten Schritt geben Sie die aktuelle Blocknummer der Versuchsperson ein.

Jede Vpn macht insgesamt 4 Blöcke: Bei Vpn mit einer Lernsitzung werden Blöcke 1-4 alle nacheinander in der ersten Sitzung gemacht. Bei Vpn mit zwei Lernsitzungen werden die Blöcke 1 und 2 in der ersten Sitzung gemacht, und die Blöcke 3 und 4 in der zweiten Sitzung. Bei Vpn mit vier Lernsitzungen wird in jeder Sitzung nur ein Block gemacht.

Wenn mehrere Blöcke nacheinander durchgeführt werden, muss nach dem Beenden eines Blocks die ***Lernapp*** neu gestartet werden, indem mit von der Startseite aus wieder auf die Lernapp klickt. Dann nochmals eingeben der Vpn-Nr, des Passworts und der nächsten Blocknummer.

Zwischen den Blöcken bei einer Sitzung können kurze Pausen gemacht werden. Bitte notieren Sie (auf der **Tabelle «Experimentdaten und Protokoll»**), ob solche gemacht wurden und wie lange diese dauerten.

2 Zweite bis vierte Lernphase / zweite bis vierte Sitzung

Dauer: 1 oder 2 Blöcke à 20 Minuten (je nach UV1)

Durchführen der nächsten Lernblöcke wie in der ersten Sitzung, je nach UV1-Zuordnung. Immer über dieselbe ***Lernapp***.

3 Testphase / zweite, dritte, oder fünfte Sitzung

Dauer: 20 Minuten

Informieren Sie die Vpn kurz, dass diese letzte Sitzung etwas anders ist als die bisherigen. In dieser werden keine neuen Wörter gelernt, sondern alle bisher gelernten abgefragt, um zu sehen, wie gut die Lernapplikation funktioniert hat. Es gibt also nur Abrufdurchgänge.

Durchführen der Testphase mit der ***Testapp***. Diese funktioniert wie die Lernapp und Sie können wie bisher dieselbe Vpn-Nr und dasselbe Passwort verwenden. Einen Link zur Testapp finden Sie ebenfalls auf der Startseite.

4 Nach den Testungen

Bewahren Sie die ausgefüllten Einverständniserklärungen und die Tabellen «Experimentdaten und Protokoll» für alle Versuchspersonen auf und geben diese am Schluss der Untersuchung an die Projektleiter ab.

Fehlerbehebung

Unterbrüche

Es kann passieren, dass die Verbindung zur Lernapp oder Testapp unterbrochen wird. Dies ist grundsätzlich nicht weiter schlimm, denn nach einem erneuten Einloggen geht es einfach dort weiter, wo man aufgehört hat; die Zeit läuft während eines Unterbruchs nicht weiter.
Stellen Sie bei einem Unterbruch sicher, dass der Browser-Tab mit der App geschlossen ist, und starten Sie dann die App neu, indem Sie von der Startseite aus auf die App klicken.
